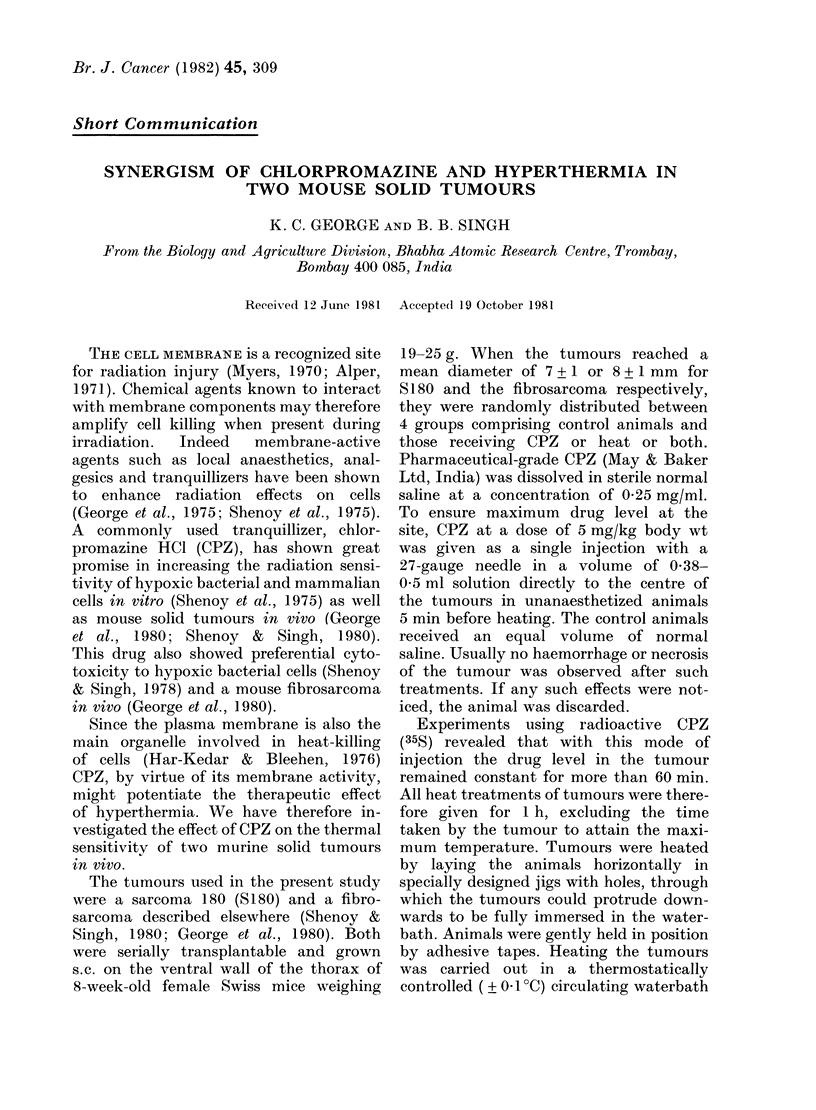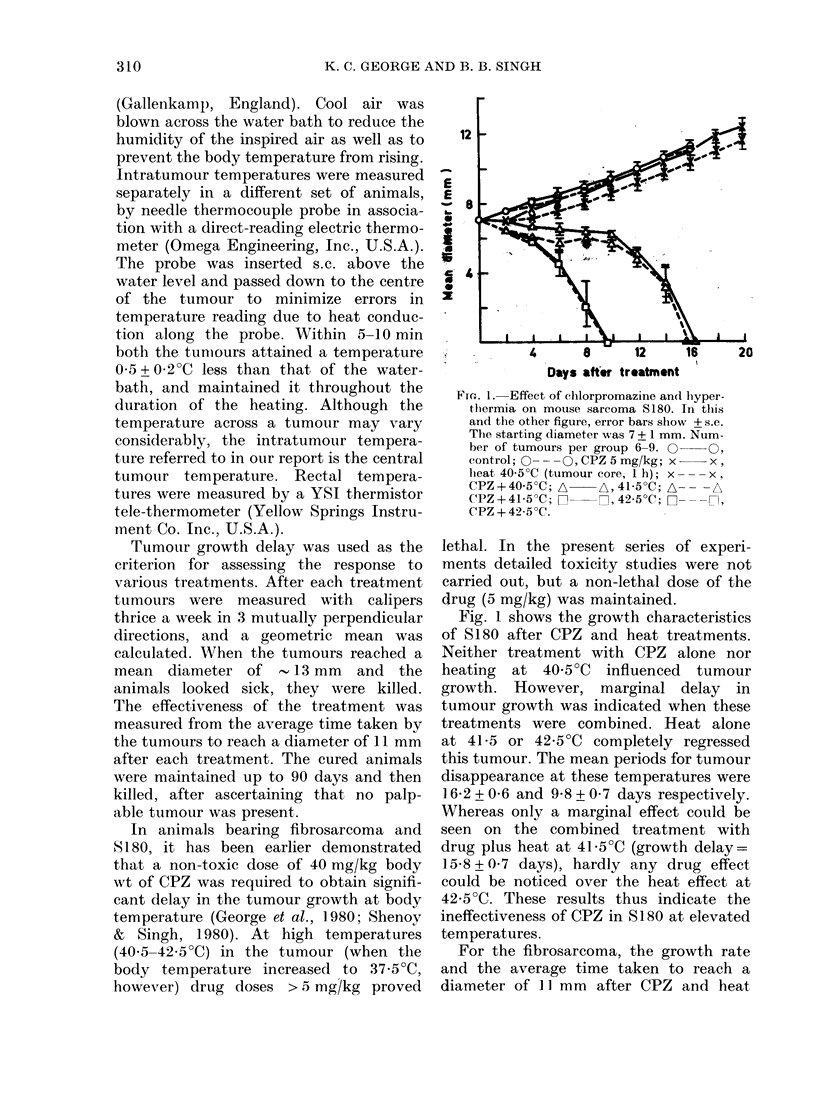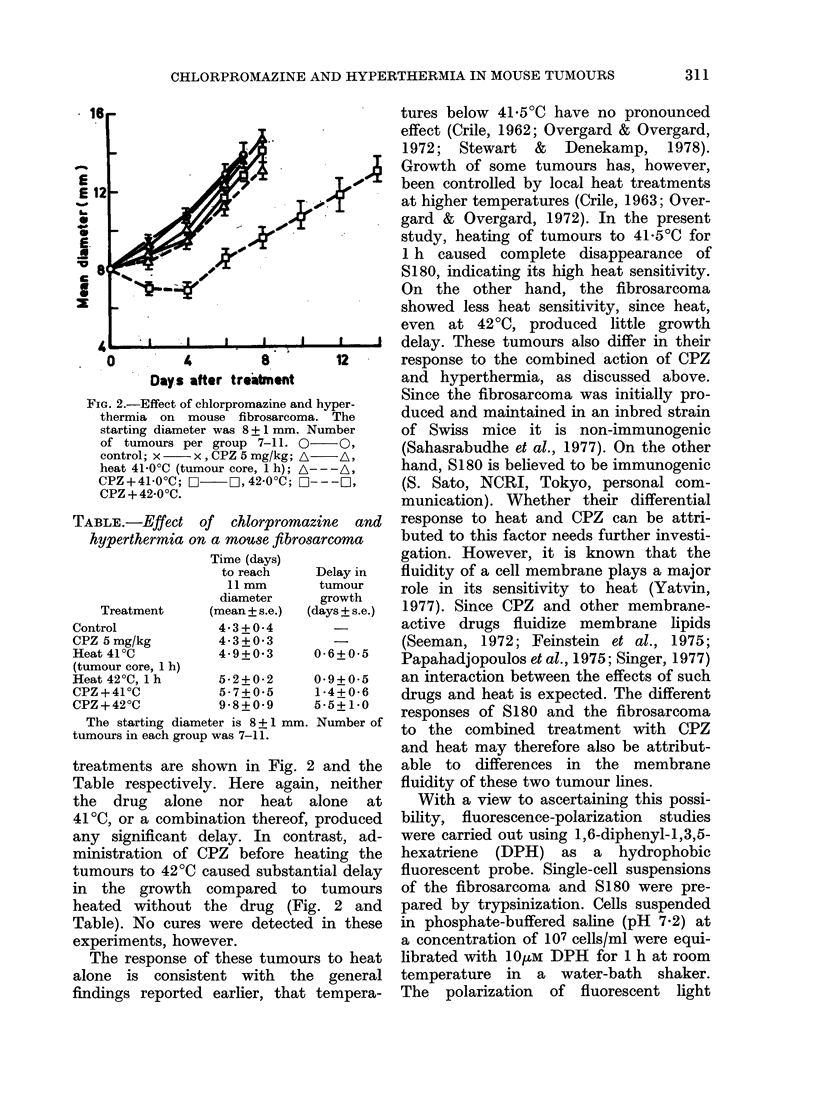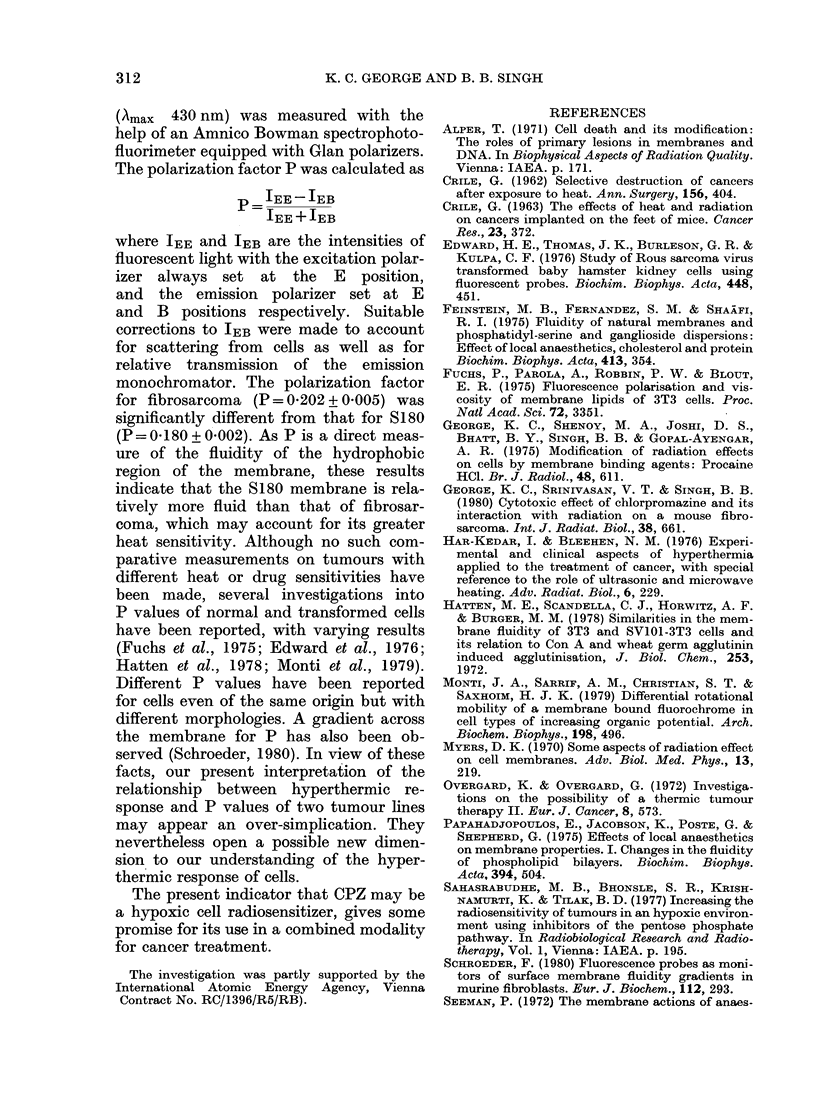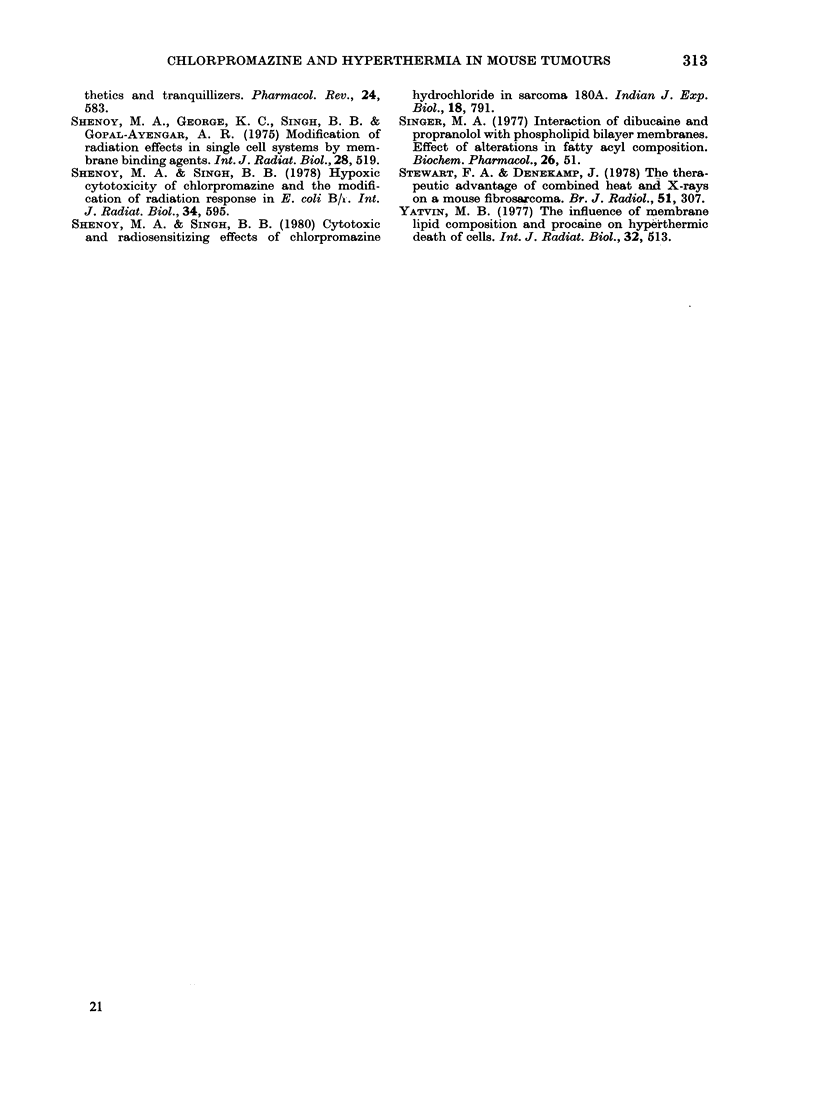# Synergism of chlorpromazine and hyperthermia in two mouse solid tumours.

**DOI:** 10.1038/bjc.1982.49

**Published:** 1982-02

**Authors:** K. C. George, B. B. Singh


*Br. J. Cancer* (1982) **45**, 309

***Short Communication***

### SYNERGISM OF CHLORPROMAZINE AND HYPERTHERMIA IN TWO MOUSE SOLID TUMOURS

K. C. GEORGE AND B. B. SINGH

*From the Biology and Agriculture Division, Bhabha Atomic Research Centre, Trombay, Bombay* 400 085, *India*

Received 12 June 1981 Accepted 19 October 1981

THE CELL MEMBRANE is a recognized site for radiation injury (Myers, 1970; Alper, 1971). Chemical agents known to interact with membrane components may therefore amplify cell killing when present during irradiation. Indeed membrane-active agents such as local anaesthetics, analgesics and tranquillizers have been shown to enhance radiation effects on cells (George *et al.*, 1975; Shenoy *et al.*, 1975). A commonly used tranquillizer, chlorpromazine HCl (CPZ), has shown great promise in increasing the radiation sensitivity of hypoxic bacterial and mammalian cells *in vitro* (Shenoy *et al.*, 1975) as well as mouse solid tumours *in vivo* (George *et al.*, 1980; Shenoy & Singh, 1980). This drug also showed preferential cytotoxicity to hypoxic bacterial cells (Shenoy & Singh, 1978) and a mouse fibrosarcoma *in vivo* (George *et al.*, 1980).

Since the plasma membrane is also the main organelle involved in heat-killing of cells (Har-Kedar & Bleehen, 1976) CPZ, by virtue of its membrane activity, might potentiate the therapeutic effect of hyperthermia. We have therefore investigated the effect of CPZ on the thermal sensitivity of two murine solid tumours *in vivo*.

The tumours used in the present study were a sarcoma 180 (S180) and a fibrosarcoma described elsewhere (Shenoy & Singh, 1980; George *et al.*, 1980). Both were serially transplantable and grown s.c. on the ventral wall of the thorax of 8-week-old female Swiss mice weighing 19–25 g. When the tumours reached a mean diameter of $7\pm1$ or $8\pm1$ mm for S180 and the fibrosarcoma respectively, they were randomly distributed between 4 groups comprising control animals and those receiving CPZ or heat or both. Pharmaceutical-grade CPZ (May & Baker Ltd, India) was dissolved in sterile normal saline at a concentration of 0·25 mg/ml. To ensure maximum drug level at the site, CPZ at a dose of 5 mg/kg body wt was given as a single injection with a 27-gauge needle in a volume of 0·38–0·5 ml solution directly to the centre of the tumours in unanaesthetized animals 5 min before heating. The control animals received an equal volume of normal saline. Usually no haemorrhage or necrosis of the tumour was observed after such treatments. If any such effects were noticed, the animal was discarded.

Experiments using radioactive CPZ ($^{35}S$) revealed that with this mode of injection the drug level in the tumour remained constant for more than 60 min. All heat treatments of tumours were therefore given for 1 h, excluding the time taken by the tumour to attain the maximum temperature. Tumours were heated by laying the animals horizontally in specially designed jigs with holes, through which the tumours could protrude downwards to be fully immersed in the waterbath. Animals were gently held in position by adhesive tapes. Heating the tumours was carried out in a thermostatically controlled ($\pm 0{\cdot}1\,°C$) circulating waterbath

(Gallenkamp, England). Cool air was blown across the water bath to reduce the humidity of the inspired air as well as to prevent the body temperature from rising. Intratumour temperatures were measured separately in a different set of animals, by needle thermocouple probe in association with a direct-reading electric thermometer (Omega Engineering, Inc., U.S.A.). The probe was inserted s.c. above the water level and passed down to the centre of the tumour to minimize errors in temperature reading due to heat conduction along the probe. Within 5–10 min both the tumours attained a temperature 0·5 ± 0·2°C less than that of the water-bath, and maintained it throughout the duration of the heating. Although the temperature across a tumour may vary considerably, the intratumour temperature referred to in our report is the central tumour temperature. Rectal temperatures were measured by a YSI thermistor tele-thermometer (Yellow Springs Instrument Co. Inc., U.S.A.).

Tumour growth delay was used as the criterion for assessing the response to various treatments. After each treatment tumours were measured with calipers thrice a week in 3 mutually perpendicular directions, and a geometric mean was calculated. When the tumours reached a mean diameter of ~13 mm and the animals looked sick, they were killed. The effectiveness of the treatment was measured from the average time taken by the tumours to reach a diameter of 11 mm after each treatment. The cured animals were maintained up to 90 days and then killed, after ascertaining that no palpable tumour was present.

In animals bearing fibrosarcoma and S180, it has been earlier demonstrated that a non-toxic dose of 40 mg/kg body wt of CPZ was required to obtain significant delay in the tumour growth at body temperature (George *et al.*, 1980; Shenoy & Singh, 1980). At high temperatures (40·5–42·5°C) in the tumour (when the body temperature increased to 37·5°C, however) drug doses > 5 mg/kg proved

Fig. 1.—Effect of chlorpromazine and hyperthermia on mouse sarcoma S180. In this and the other figure, error bars show ± s.e. The starting diameter was 7 ± 1 mm. Number of tumours per group 6–9. ○——○, control; ○– – –○, CPZ 5 mg/kg; ×——×, heat 40·5°C (tumour core, 1 h); ×– – –×, CPZ + 40·5°C; △——△, 41·5°C; △– – –△ CPZ + 41·5°C; □——□, 42·5°C; □– – –□, CPZ + 42·5°C.

lethal. In the present series of experiments detailed toxicity studies were not carried out, but a non-lethal dose of the drug (5 mg/kg) was maintained.

Fig. 1 shows the growth characteristics of S180 after CPZ and heat treatments. Neither treatment with CPZ alone nor heating at 40·5°C influenced tumour growth. However, marginal delay in tumour growth was indicated when these treatments were combined. Heat alone at 41·5 or 42·5°C completely regressed this tumour. The mean periods for tumour disappearance at these temperatures were 16·2 ± 0·6 and 9·8 ± 0·7 days respectively. Whereas only a marginal effect could be seen on the combined treatment with drug plus heat at 41·5°C (growth delay = 15·8 ± 0·7 days), hardly any drug effect could be noticed over the heat effect at 42·5°C. These results thus indicate the ineffectiveness of CPZ in S180 at elevated temperatures.

For the fibrosarcoma, the growth rate and the average time taken to reach a diameter of 11 mm after CPZ and heat

FIG. 2.—Effect of chlorpromazine and hyperthermia on mouse fibrosarcoma. The starting diameter was 8 ± 1 mm. Number of tumours per group 7–11. ○——○, control; ×——×, CPZ 5 mg/kg; △——△, heat 41·0°C (tumour core, 1 h); △– – –△, CPZ + 41·0°C; □——□, 42·0°C; □– – –□, CPZ + 42·0°C.

TABLE.—*Effect of chlorpromazine and hyperthermia on a mouse fibrosarcoma*

| Treatment | Time (days) to reach 11 mm diameter (mean ± s.e.) | Delay in tumour growth (days ± s.e.) |
|---|---|---|
| Control | 4·3 ± 0·4 | — |
| CPZ 5 mg/kg | 4·3 ± 0·3 | — |
| Heat 41°C (tumour core, 1 h) | 4·9 ± 0·3 | 0·6 ± 0·5 |
| Heat 42°C, 1 h | 5·2 ± 0·2 | 0·9 ± 0·5 |
| CPZ + 41°C | 5·7 ± 0·5 | 1·4 ± 0·6 |
| CPZ + 42°C | 9·8 ± 0·9 | 5·5 ± 1·0 |

The starting diameter is 8 ± 1 mm. Number of tumours in each group was 7–11.

treatments are shown in Fig. 2 and the Table respectively. Here again, neither the drug alone nor heat alone at 41 °C, or a combination thereof, produced any significant delay. In contrast, administration of CPZ before heating the tumours to 42 °C caused substantial delay in the growth compared to tumours heated without the drug (Fig. 2 and Table). No cures were detected in these experiments, however.

The response of these tumours to heat alone is consistent with the general findings reported earlier, that temperatures below 41·5°C have no pronounced effect (Crile, 1962; Overgard & Overgard, 1972; Stewart & Denekamp, 1978). Growth of some tumours has, however, been controlled by local heat treatments at higher temperatures (Crile, 1963; Overgard & Overgard, 1972). In the present study, heating of tumours to 41·5°C for 1 h caused complete disappearance of S180, indicating its high heat sensitivity. On the other hand, the fibrosarcoma showed less heat sensitivity, since heat, even at 42°C, produced little growth delay. These tumours also differ in their response to the combined action of CPZ and hyperthermia, as discussed above. Since the fibrosarcoma was initially produced and maintained in an inbred strain of Swiss mice it is non-immunogenic (Sahasrabudhe *et al.*, 1977). On the other hand, S180 is believed to be immunogenic (S. Sato, NCRI, Tokyo, personal communication). Whether their differential response to heat and CPZ can be attributed to this factor needs further investigation. However, it is known that the fluidity of a cell membrane plays a major role in its sensitivity to heat (Yatvin, 1977). Since CPZ and other membrane-active drugs fluidize membrane lipids (Seeman, 1972; Feinstein *et al.*, 1975; Papahadjopoulos *et al.*, 1975; Singer, 1977) an interaction between the effects of such drugs and heat is expected. The different responses of S180 and the fibrosarcoma to the combined treatment with CPZ and heat may therefore also be attributable to differences in the membrane fluidity of these two tumour lines.

With a view to ascertaining this possibility, fluorescence-polarization studies were carried out using 1,6-diphenyl-1,3,5-hexatriene (DPH) as a hydrophobic fluorescent probe. Single-cell suspensions of the fibrosarcoma and S180 were prepared by trypsinization. Cells suspended in phosphate-buffered saline (pH 7·2) at a concentration of $10^7$ cells/ml were equilibrated with 10$\mu$M DPH for 1 h at room temperature in a water-bath shaker. The polarization of fluorescent light

($\lambda_{max}$ 430 nm) was measured with the help of an Amnico Bowman spectrophotofluorimeter equipped with Glan polarizers. The polarization factor P was calculated as

$$P = \frac{I_{EE} - I_{EB}}{I_{EE} + I_{EB}}$$

where $I_{EE}$ and $I_{EB}$ are the intensities of fluorescent light with the excitation polarizer always set at the E position, and the emission polarizer set at E and B positions respectively. Suitable corrections to $I_{EB}$ were made to account for scattering from cells as well as for relative transmission of the emission monochromator. The polarization factor for fibrosarcoma ($P = 0{\cdot}202 \pm 0{\cdot}005$) was significantly different from that for S180 ($P = 0{\cdot}180 \pm 0{\cdot}002$). As P is a direct measure of the fluidity of the hydrophobic region of the membrane, these results indicate that the S180 membrane is relatively more fluid than that of fibrosarcoma, which may account for its greater heat sensitivity. Although no such comparative measurements on tumours with different heat or drug sensitivities have been made, several investigations into P values of normal and transformed cells have been reported, with varying results (Fuchs *et al.*, 1975; Edward *et al.*, 1976; Hatten *et al.*, 1978; Monti *et al.*, 1979). Different P values have been reported for cells even of the same origin but with different morphologies. A gradient across the membrane for P has also been observed (Schroeder, 1980). In view of these facts, our present interpretation of the relationship between hyperthermic response and P values of two tumour lines may appear an over-simplication. They nevertheless open a possible new dimension to our understanding of the hyperthermic response of cells.

The present indicator that CPZ may be a hypoxic cell radiosensitizer, gives some promise for its use in a combined modality for cancer treatment.

The investigation was partly supported by the International Atomic Energy Agency, Vienna Contract No. RC/1396/R5/RB).

### REFERENCES

ALPER, T. (1971) Cell death and its modification: The roles of primary lesions in membranes and DNA. In *Biophysical Aspects of Radiation Quality*. Vienna: IAEA. p. 171.

CRILE, G. (1962) Selective destruction of cancers after exposure to heat. *Ann. Surgery*, **156**, 404.

CRILE, G. (1963) The effects of heat and radiation on cancers implanted on the feet of mice. *Cancer Res.*, **23**, 372.

EDWARD, H. E., THOMAS, J. K., BURLESON, G. R. & KULPA, C. F. (1976) Study of Rous sarcoma virus transformed baby hamster kidney cells using fluorescent probes. *Biochim. Biophys. Acta*, **448**, 451.

FEINSTEIN, M. B., FERNANDEZ, S. M. & SHAĀFI, R. I. (1975) Fluidity of natural membranes and phosphatidyl-serine and ganglioside dispersions: Effect of local anaesthetics, cholesterol and protein *Biochim. Biophys. Acta*, **413**, 354.

FUCHS, P., PAROLA, A., ROBBIN, P. W. & BLOUT, E. R. (1975) Fluorescence polarisation and viscosity of membrane lipids of 3T3 cells. *Proc. Natl Acad. Sci.* **72**, 3351.

GEORGE, K. C., SHENOY, M. A., JOSHI, D. S., BHATT, B. Y., SINGH, B. B. & GOPAL-AYENGAR, A. R. (1975) Modification of radiation effects on cells by membrane binding agents: Procaine HCl. *Br. J. Radiol.*, **48**, 611.

GEORGE, K. C., SRINIVASAN, V. T. & SINGH, B. B. (1980) Cytotoxic effect of chlorpromazine and its interaction with radiation on a mouse fibrosarcoma. *Int. J. Radiat. Biol.*, **38**, 661.

HAR-KEDAR, I. & BLEEHEN, N. M. (1976) Experimental and clinical aspects of hyperthermia applied to the treatment of cancer, with special reference to the role of ultrasonic and microwave heating. *Adv. Radiat. Biol.*, **6**, 229.

HATTEN, M. E., SCANDELLA, C. J., HORWITZ, A. F. & BURGER, M. M. (1978) Similarities in the membrane fluidity of 3T3 and SV101-3T3 cells and its relation to Con A and wheat germ agglutinin induced agglutinisation, *J. Biol. Chem.*, **253**, 1972.

MONTI, J. A., SARRIF, A. M., CHRISTIAN, S. T. & SAXHOIM, H. J. K. (1979) Differential rotational mobility of a membrane bound fluorochrome in cell types of increasing organic potential. *Arch. Biochem. Biophys.*, **198**, 496.

MYERS, D. K. (1970) Some aspects of radiation effect on cell membranes. *Adv. Biol. Med. Phys.*, **13**, 219.

OVERGARD, K. & OVERGARD, G. (1972) Investigations on the possibility of a thermic tumour therapy II. *Eur. J. Cancer*, **8**, 573.

PAPAHADJOPOULOS, E., JACOBSON, K., POSTE, G. & SHEPHERD, G. (1975) Effects of local anaesthetics on membrane properties. I. Changes in the fluidity of phospholipid bilayers. *Biochim. Biophys. Acta*, **394**, 504.

SAHASRABUDHE, M. B., BHONSLE, S. R., KRISHNAMURTI, K. & TILAK, B. D. (1977) Increasing the radiosensitivity of tumours in an hypoxic environment using inhibitors of the pentose phosphate pathway. In *Radiobiological Research and Radiotherapy*, Vol. 1, Vienna: IAEA. p. 195.

SCHROEDER, F. (1980) Fluorescence probes as monitors of surface membrane fluidity gradients in murine fibroblasts. *Eur. J. Biochem.*, **112**, 293.

SEEMAN, P. (1972) The membrane actions of anaesthetics and tranquillizers. *Pharmacol. Rev.*, **24**, 583.

SHENOY, M. A., GEORGE, K. C., SINGH, B. B. & GOPAL-AYENGAR, A. R. (1975) Modification of radiation effects in single cell systems by membrane binding agents. *Int. J. Radiat. Biol.*, **28**, 519.

SHENOY, M. A. & SINGH, B. B. (1978) Hypoxic cytotoxicity of chlorpromazine and the modification of radiation response in *E. coli* B/r. *Int. J. Radiat. Biol.*, **34**, 595.

SHENOY, M. A. & SINGH, B. B. (1980) Cytotoxic and radiosensitizing effects of chlorpromazine hydrochloride in sarcoma 180A. *Indian J. Exp. Biol.*, **18**, 791.

SINGER, M. A. (1977) Interaction of dibucaine and propranolol with phospholipid bilayer membranes. Effect of alterations in fatty acyl composition. *Biochem. Pharmacol.*, **26**, 51.

STEWART, F. A. & DENEKAMP, J. (1978) The therapeutic advantage of combined heat and X-rays on a mouse fibrosarcoma. *Br. J. Radiol.*, **51**, 307.

YATVIN, M. B. (1977) The influence of membrane lipid composition and procaine on hyperthermic death of cells. *Int. J. Radiat. Biol.*, **32**, 513.